# Introducing the CSP Analyzer: A novel Machine Learning-based application for automated analysis of two-dimensional NMR spectra in NMR fragment-based screening

**DOI:** 10.1016/j.csbj.2020.02.015

**Published:** 2020-02-28

**Authors:** R. Fino, R. Byrne, C.A. Softley, M. Sattler, G. Schneider, G.M. Popowicz

**Affiliations:** a Institute of Structural Biology, Helmholtz Zentrum München, Neuherberg, Germany; b Biomolecular NMR, Bayerisches NMR Zentrum and Center for Integrated Protein Science Munich at Chemistry Department, Technical University of Munich, Garching, Germany; c Department of Chemistry and Applied Biosciences, Institute of Pharmaceutical Sciences, Swiss Federal Institute of Technology (ETH), Vladimir-Prelog-Weg 4, 8093 Zürich, Switzerland

**Keywords:** 2-D NMR, Fragment screening, Machine-learning, Automatic CSP analysis, C# GUI, Fragment-based drug discovery

## Abstract

NMR-based screening, especially fragment-based drug discovery is a valuable approach in early-stage drug discovery. Monitoring fragment-binding in protein-detected 2D NMR experiments requires analysis of hundreds of spectra to detect chemical shift perturbations (CSPs) in the presence of ligands screened. Computational tools are available that simplify the tracking of CSPs in 2D NMR spectra. However, to the best of our knowledge, an efficient automated tool for the assessment and binning of multiple spectra for ligand binding has not yet been described. We present a novel and fast approach for analysis of multiple 2D HSQC spectra based on machine-learning-driven statistical discrimination. The CSP Analyzer features a C# frontend interfaced to a Python ML classifier. The software allows rapid evaluation of 2D screening data from large number of spectra, reducing user-introduced bias in the evaluation. The CSP Analyzer software package is available on GitHub https://github.com/rubbs14/CSP-Analyzer/releases/tag/v1.0 under the GPL license 3.0 and is free to use for academic and commercial uses.

## Graphical abstract

**Figure:**
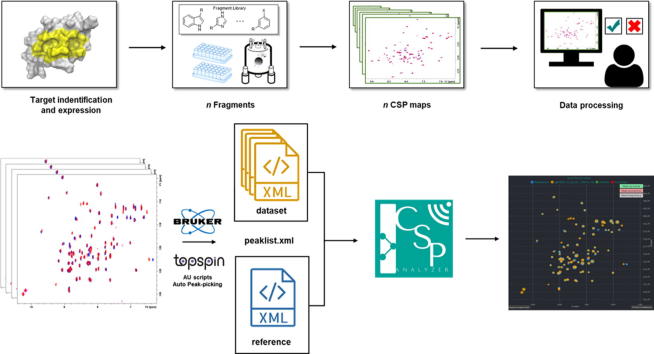

## 1 Introduction

In recent times, fragment-based drug discovery (FBDD) has become progressively more important in early-stage drug design. Fragment-based screening (FBS) offers an efficient, rational, way to find small molecule inhibitors. By testing for binding of small fragments, a large chemical space can be tested with fewer molecules than with other approaches. This is due to the higher probability of a suitable binding pocket or position being present with lower complexity molecules [1], leading to higher efficiency (both screening efficiency and ligand efficiency), and elucidating possible starting points for further drug discovery. The binding sites of these fragments can then be located on the surface of the protein and from there, fragments connected or grown to maximize interactions with the surrounding area, eventually leading to hit and lead molecules. X-ray crystallography and nuclear magnetic resonance (NMR) can be used to both identify hits and get structural information about the binding. NMR, working in the solution state, and requiring low concentrations, is well placed to detect weakly-binding fragments, in an environment more representative of in vivo conditions. Since the turn of the century [2], FBS has grown in use, in both academic and industrial settings, owing to its speed and reliability at testing large chemical space with standard and cost-effective biophysical methods. As such, thousands-strong libraries can be quickly measured against a huge variety of biological targets.

With NMR, both target- and ligand-observed screening approaches are possible [3], [4], [5], [6], [7], [8]. From a technical point of view, NMR FBS allows medium to large libraries of fragments (1000–5000 fragments) to be efficiently screened. Concerning binding affinity ranges, FBS by NMR allows detecting binding of ligands with affinities as weak as low millimolar [2]. Furthermore, certain experiments may provide helpful insights into the kinetics and dynamics of fragment binding [9].

Ligand-observed NMR screening utilizes simple and sensitive 1D experiments (i.e. STD [8], WaterLOGSY [10], SLAPSTIC [11], T2 and T1rho [12]) and requires no isotopic-labeling of the target protein, simplifying the experimental preparation. These ligand-observed NMR experiments are applicable to medium to very high molecular-weight targets. Depending on the ligand affinity, direct or competitive binding is monitored [8], [9], [13].

For smaller proteins (<30 kDa), protein-observed 2D heteronuclear NMR experiments, such as ^1^H, ^15^N correlation (HSQC [14], SOFAST-HMQC [15]) are also used [16]. This requires isotope labeling of the target protein – often ^15^N or ^13^C alone for small proteins or combined with ^2^H-labeling [17] for larger proteins [18]. As STD and T1rho, for example, work better with larger proteins as a result of slower tumbling, 2D experiments are especially preferred with small proteins as a reliable procedure that measures the direct effect on protein chemical environment, rather than transferred magnetization or change in ligand properties. It has the additional advantage, in cases where the amino acid shifts are assigned, that it gives an indication of possible binding regions by means of the peaks’ chemical shift perturbations (CSP) [19], [20], [21].

Automated analysis tools are available for 1D NMR experiments [22], [23], [24] and 2D NMR experiments [25], [26], [27]. These tools allow the analysis of 2D NMR titration experiments by tracking of CSP. Following titration assignments in order to determine rate constants as in NvMap [25], however, is quite different to the needs of fragment screening, which requires a more global view of whether the overall spectrum is significantly altered. In practice, this means that a significant number of peaks alter position or intensity or broaden upon addition of the fragment or ligand in question. Each spectrum measured with a fragment is overlaid with the reference spectrum and compared directly. This is easily done by eye, but, depending on the number of NMR spectra recorded in an FBS campaign, manual analysis can be time-consuming. The other significant issue with manual analysis is the addition of human bias. Whether as a result of tiredness, splitting the analysis over multiple sessions, or simply as a result of comparison with those already considered, the same spectrum can be classified differently depending on its position within the dataset. Automation or partial automation of this process would improve efficiency and accuracy, as well as reducing opportunities for the introduction of human bias. It would enable direct comparisons of achieved hit rates with different proteins, as currently these campaigns are often analyzed by different people, leading to differences in subjective evaluation of the spectra. As far as we are aware, fast and reliable automated analysis software for the simultaneous analysis of large numbers of 2D NMR spectra that provides useful binning for hit identification has not been reported in the literature.

In this work, we present a novel approach for very fast analysis of hundreds of 2D NMR spectra in FBS based on advanced machine-learning-driven statistical discrimination. Our CSP Analyzer features a C# frontend that is interfaced with a Python Machine Learning classifier.

According to the principles of target-based NMR 2D FBS, the software is designed to identify the “active” fragments by comparing each spectrum (protein with fragment or fragment pool) in a screening set to its reference spectrum (protein only). This operation does not require the assignment of the peaks because the statistics are based on the fingerprinting of each spectrum properties (peaks position, peaks scattering, intensities, etc.). In other words, the algorithms included in the package are capable of indicating, with good recall, the “most different” experiments to the reference spectrum. The software is designed to enable either partial or fully automated hit determination from measured 2-D spectra, depending on the user’s requirements, thus being a versatile tool for FBS campaigns by NMR.

## 2 Results

To train the machine-learning (ML) model, we focused on developing an input representation, borrowing heavily from advances in computer vision, to enable multi-protein spectral analysis without retraining for each new target of interest. We optimized our architecture for the particulars of this challenge, specifically recognizing the relative importance, and rarity, of ‘active’ vs. ‘inactive’ spectra. To accomplish these objectives, we adopted a mixture of strategies, engineering a useful feature representation that draws on computer vision methodology, and through synthetic data enhancement, weighting, and hyperparameter optimization.

We tested the program and the ML discriminator on our in-house datasets of NMR-based fragment screening of four different protein targets. We refer in this work to the “active” spectra as the spectra of those fragments that cause CSP, when compared to the protein reference spectrum for each dataset. On the other hand, the “inactive” spectra are the spectra generated by fragments that are not interacting with the protein, thus not causing any noticeable CSP. A “broken” spectrum is defined when problems in locking or shimming of the samples caused unreadable or very noisy spectra.

Spectral CSP plots were generated using the TopSpin 3.2 (Bruker) automatic peak picking algorithm and exported as XML files for each experiment, keeping the minimum intensity contour threshold limits as the intensity of the reference spectrum in the dataset. The limits for the F1 and F2 dimensions of the spectra were also set in order to exclude the noise due to the water signal in the F1 region (lower limit higher than 6 ppm) in order to avoid peak picking of the noise signals. While this approach is subject to the accuracy of the peak picking capabilities of TopSpin software, we decided to use this method to test our proof-of-concept ML-based discriminator.

**Fig. 1:**
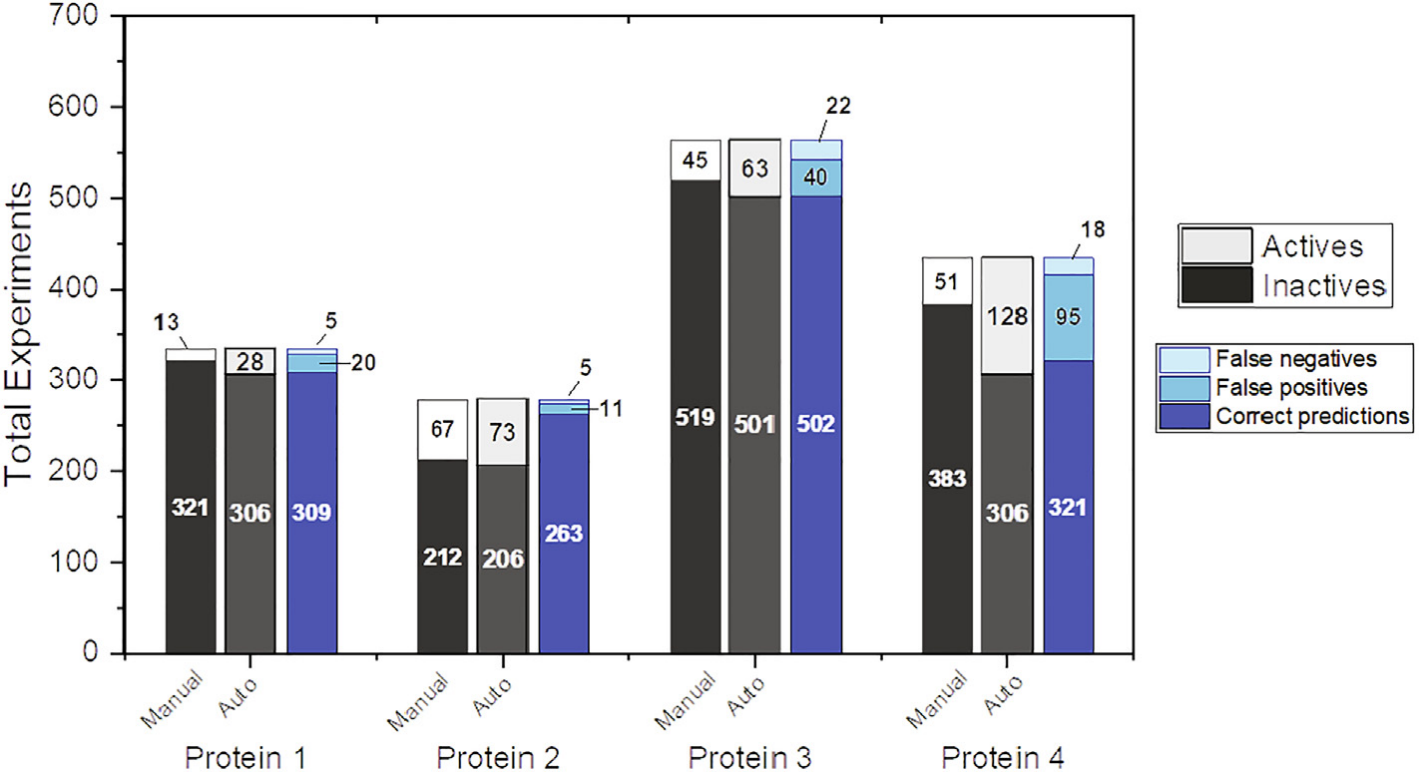
Machine Learning-based discriminator results for protein datasets. In Figure is reported the overall performance of the ML analysis. Correct predictions refer to the number of experiments that were identified correctly by the ML-based discriminator, as compared to the user selection (marked as “active” or “inactive”, depending on the CSPs). False positives refer to those experiments that were predicted as “inactive” while the manual user marked them as “active”. False negatives refer to the number of experiments that were marked as “active” by the ML-based discriminator but marked as “inactive” by the user. The results displayed are referred to FBS campaigns against four different proteins. For each protein, the first stacked column represents the distribution of the experiments after manual selection; the second column summarizes the results of the automated analysis. The third stacked column shows the distribution of the statistics of the discriminator, reporting for each protein the number of correct predictions, false positives and false negatives in the corresponding datasets.

We trained the ML model using randomly-picked experiments from all four protein datasets which were previously processed by expert NMR spectroscopists. The total set of experiments available for analysis consisted of 1611 2-D HSQC NMR spectra recorded after screening our in-house library of 1500 fragments. The full dataset contained both spectra recorded using cocktail pools of fragments, as well as single ligand pools used for deconvolution. The screening campaigns were conducted against four different protein targets. A total number of 32 active spectra were then mixed with randomly-picked inactive and noisy spectra coming from each of the available protein datasets, creating training sets consisting of 100 spectra (6.2% of the total number of spectra). The aim of the mixed sampling was to avoid overfitting of the model and avoid introducing a bias toward one of the datasets with the highest amount of active spectra. We then trained the discriminator using a training set of randomly-picked selections from active and inactive spectra in all the datasets. Validation was then performed on all the available experiments. Even with a small training set, the algorithm was capable of identifying the spectra correctly with good efficiency (Fig. 1).

The relatively high number of false positives identified by the ML analysis is due to the fact that the algorithm was designed in order to include all the spectral plots that are consistently different from the given reference spectrum. While this approach impacts negatively on the overall accuracy of the predictions, it keeps the number of false negatives as low as possible. Ultimately, the purpose of this method is to avoid the case that potentially active compounds are not identified and reported to the user during the FBS campaign data processing.

On the other hand, noisy spectra or partially failed experiments may be reported as actives when they are not. In light of this, the parameters chosen for peak picking in TopSpin are crucial for a correct analysis. Protein 4 is an example of a dataset containing experiments with very variable background noise and low-intensity local peak clustering. Due to this behavior, the identification of active pools for this specific target represented a challenging task even for the human experimentalist. Nevertheless, the ML analysis was capable of recalling the interesting experiments keeping the false-negative rate low, thus confirming its robustness and efficiency.

With an average model accuracy of 0.87, we demonstrated that the ML-discriminator can be used to assist in the rapid processing of hundreds of spectra, whilst maintaining good reliability.

Furthermore, with the graphical user interface (GUI) of the CSP Analyzer, which is designed to handle, display and process several hundred spectra as efficiently as possible, the user can easily keep track of the status of the analysis and get an overall estimation of the dataset quality. At the end of the analysis, all the data can be exported and saved either as a PDF report or as an Excel spreadsheet.

## 3 Frontend

### 3.1 GUIi

**Fig. 2:**
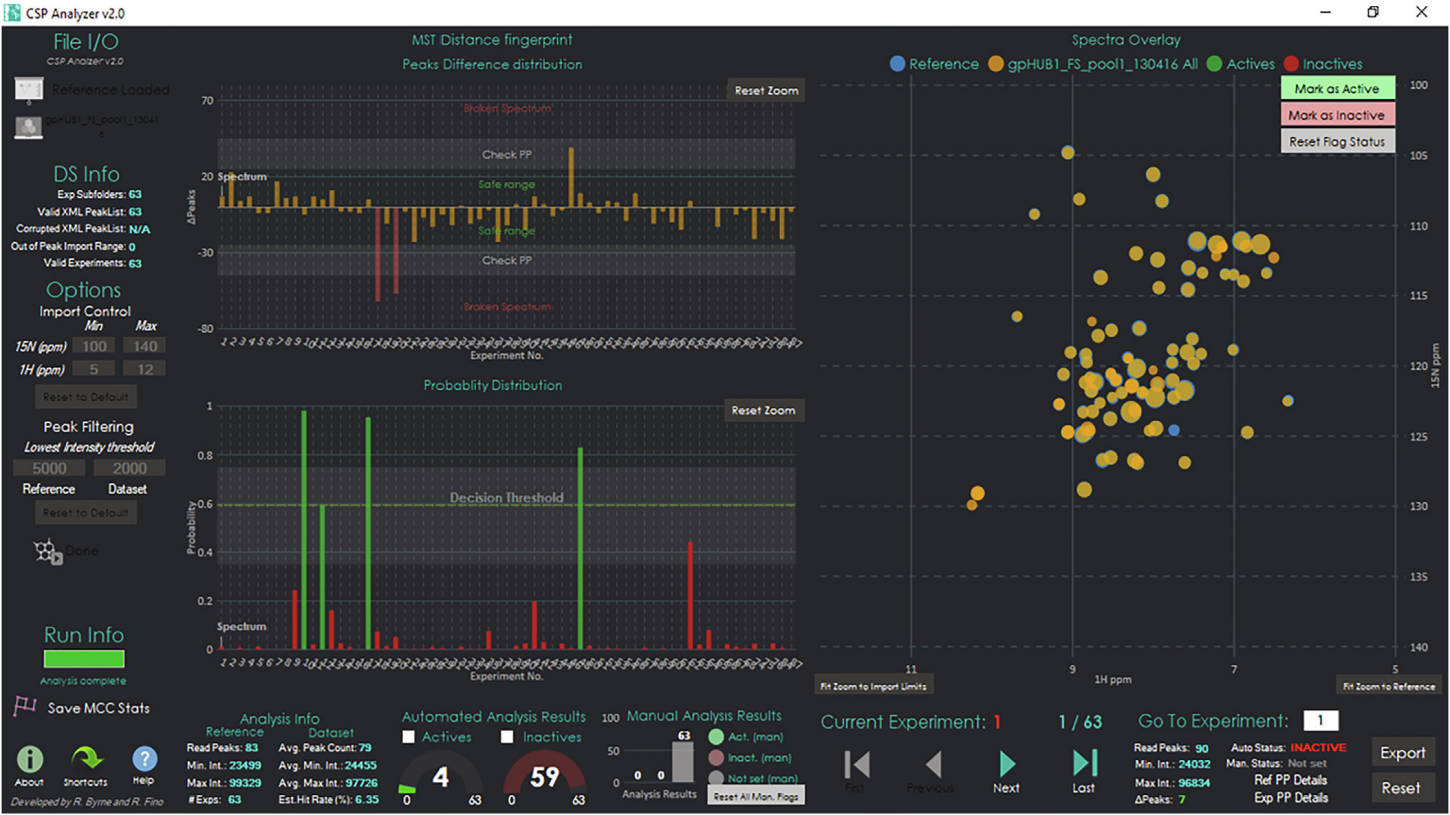
GUI of the CSP Analyzer.

The GUI for the CSP analyzer (Fig. 2) is written in C# using the .NET Framework 4.6 available in Microsoft Visual Studio 2017 Community Edition. Plots and graphs use the LiveCharts 0.9.1 libraries [28].

The user can browse through folders, select and load the XML file for the reference spectrum in the dataset. A custom lower-bound intensity threshold high-pass filter can be set independently for the reference and for the dataset in order to deal with eventual noisy peak picking. Once the reference is loaded, the spectral plot XML files for the desired dataset can be loaded at once by selecting a folder that contains all of the files. Only experiments that contain the proper “peaklist.xml” files will be displayed and overlaid with the reference spectrum. A message will display a log of the loading process, reporting back to the user in the event of an empty folder (i.e. failed experiment).

**Fig. 3:**
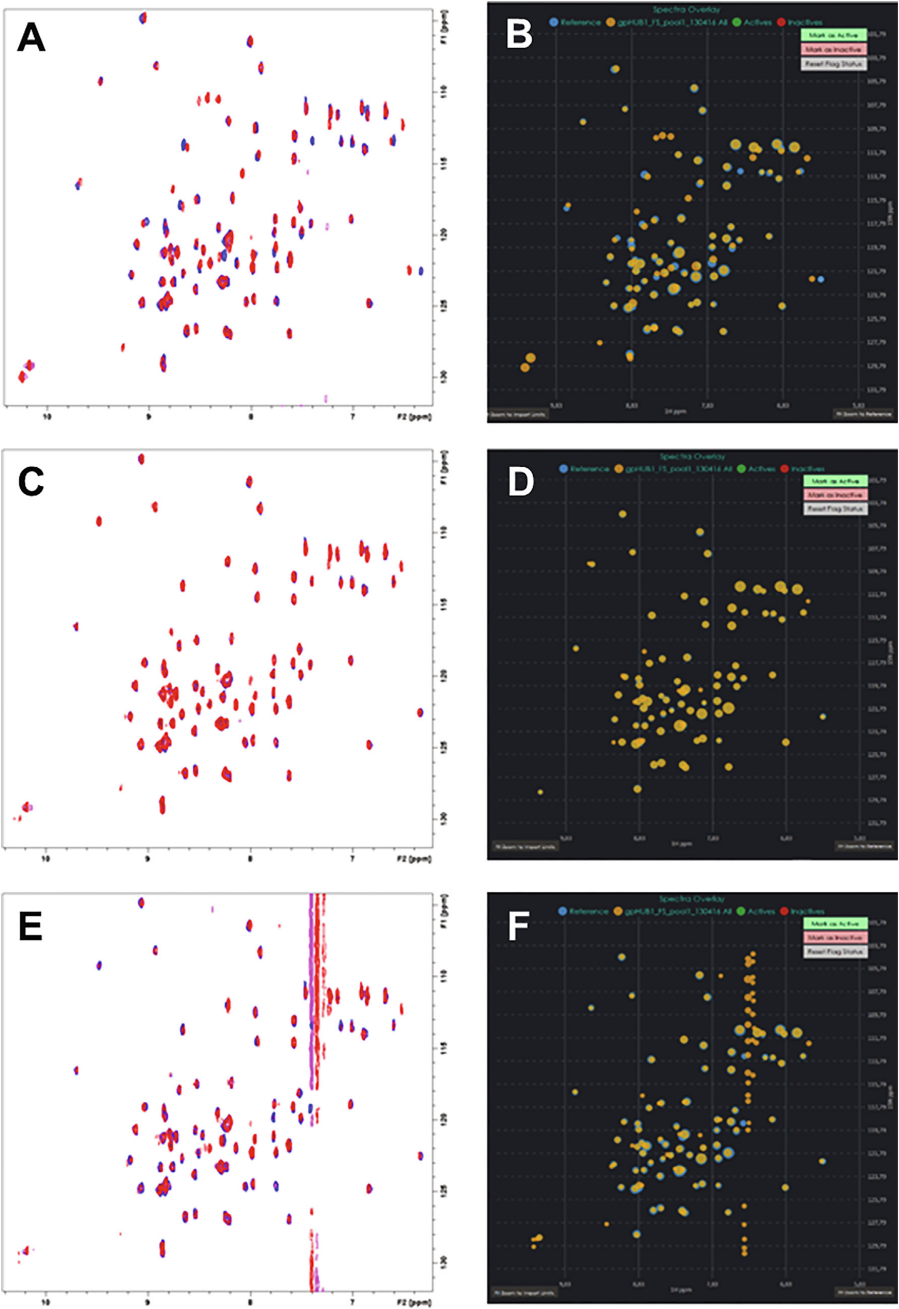
Side-by-side comparison of CSP maps for experiments marked as active (A, B), inactive (C, D) and noisy or broken spectrum (E, F). For reader reference: TopSpin visualization is always shown in the left panel, while CSP Analyzer rendering results are always shown in the right panel. The diameter of the peaks in the CSP Analyzer rendering corresponds to the absolute intensity of the peak in the output XML file after TopSpin peak picking. After mouse-over on a peak, its coordinates and the intensity will be shown as a popup message. If the automatic analysis is performed, the spectra can be clustered by their activity and the user can cycle to the selected subset only from the respective panel in the GUI.

The user interface displays useful information about the NMR screening data, such as the total number of peaks in both the reference spectrum and the dataset spectrum, the peak number difference between the overlaid experiments, the minimum and the maximum intensity, the average minimum and maximum intensities in the dataset and the average number of peaks in the dataset experiments. Fig. 3 shows a side-by-side comparison of the spectra rendering for experiments marked respectively as active, inactive and broken or noisy spectrum from the processed datasets used for validation and analysis.

A control module can be used to skim quickly through the dataset experiments. A go-to function allows quick navigation to the selected experiment.

**Fig. 4:**
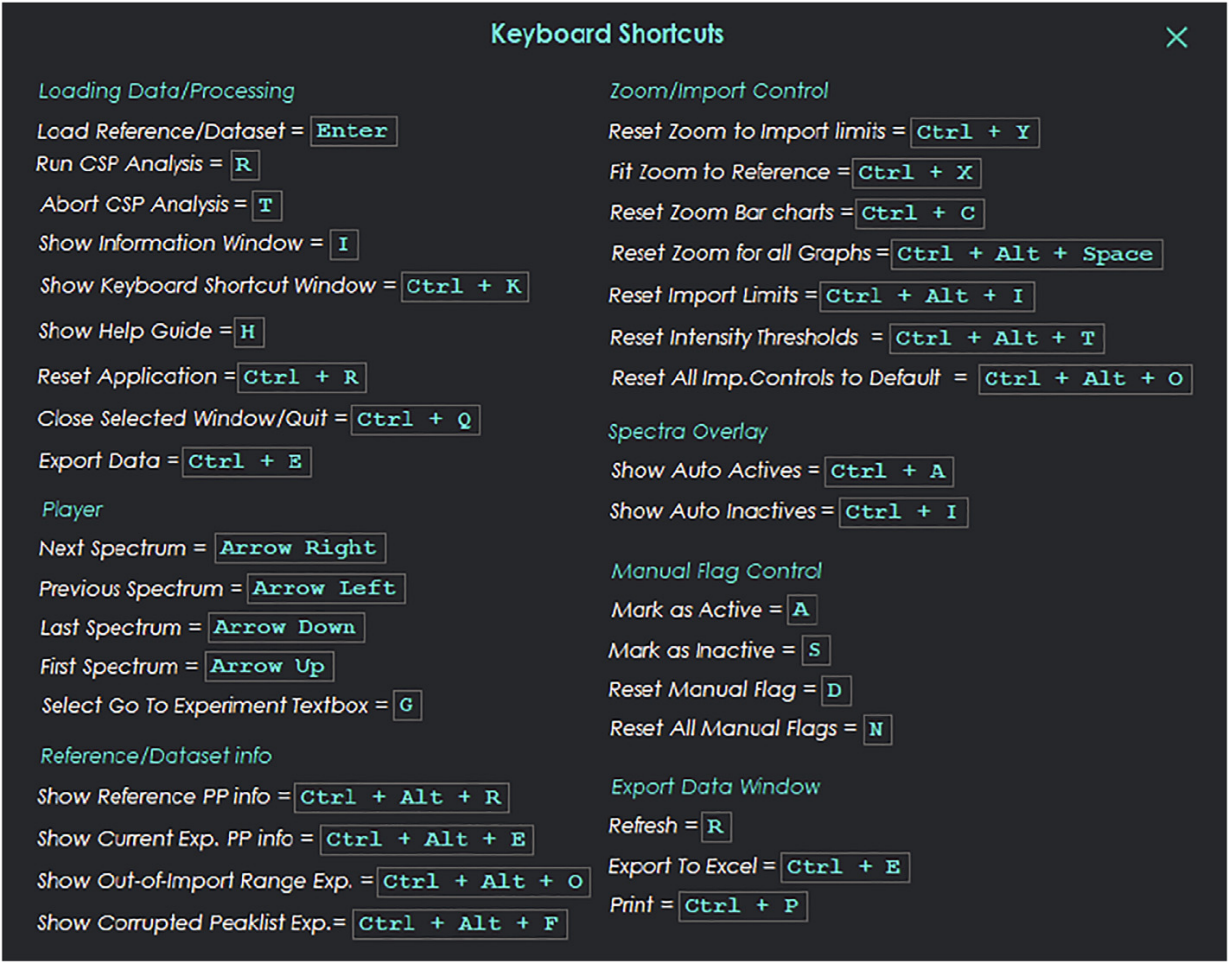
A list of available keyboard shortcuts for the CSP Analyzer GUI.

All the plots are fully zoomable and the probability and peak difference distribution bar plots are also clickable. Customized keyboard shortcuts are available in order to facilitate the use of the program (Fig. 4).

Furthermore, clicking on the “Help” button, the user can access a troubleshooting guide for known issues (such as memory exceptions that may be caused by the Conda environment distributed with the software package) and some guidelines to optimize peak picking in TopSpin. To this purpose, a string-generator module can be used to easily generate command-line strings with the proper syntax required by TopSpin peak-picking automation.

Before running the ML-backend discriminator for analysis, the software converts the spectra loaded according to the noise-filter to JSON strings using the Newtonsoft JSON serializer libraries available for C# .NET framework. [29] The processed JSON may be also be saved to a custom path for further processing (i.e. for training purposes). After the analysis, the user can selectively display the subsets marked as “active” or as “inactive” by the ML-discriminator and use the player module to skim through the desired experiments. A list of keyboard shortcuts can be displayed by clicking on the corresponding button. The user can mark a spectrum in the dataset manually as “active” or “inactive” using the respective buttons in the overlay area. The status can be reset at any time using the respective button.

**Fig. 5:**
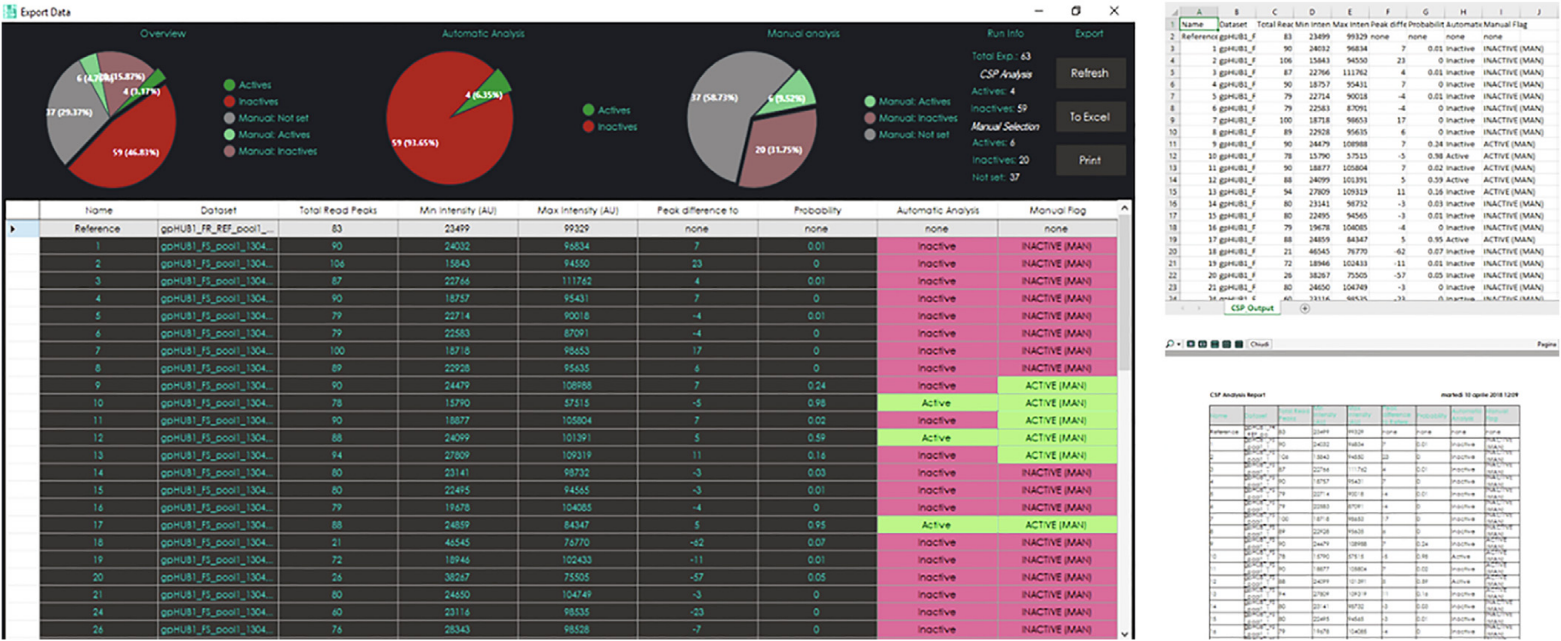
Export window with printing preview and a sample dataset exported to Excel.

Clicking the “Export” button a new window (Fig. 5) with a detailed overview of the analysis data will be shown; the user can choose if print the analysis output (either sending the job to an installed printer or to PDF) or export the data analysis to Excel for further processing.

Distributed with the software package is provided a separate program, named PeakListExtractor.exe, that can be used to retrieve only the peak lists generated after peak picking preserving the same folder-tree structure used by TopSpin, thus avoiding to copy the full experiments data on the local drive. For the ease of deployability for this and future versions of the CSP Analyzer, we decided to build a Miniconda environment (https://docs.conda.io/en/latest/miniconda.html) containing the framework and the libraries required for the ML discriminator which is distributed with the software release.

The software package can be downloaded from GitHub https://github.com/rubbs14/CSP-Analyzer/releases/tag/v1.0 as a Visual Studio C# project, which can then be compiled to generate the binaries (among with the Miniconda3 and the ML framework) needed to run the program. The project can be also forked from the original GitHub repository. A demo dataset for testing the correct installation is provided alongside the build binaries and is located in the folder from where the software is launched.

## 4 Backend

### 4.1 Computational assessment

Whilst in the early versions of the software we approached the active identification issue using a purely analytical method based on local distance calculation using a customized version of the Hungarian algorithm (HA) [30], this approach was seen to give an high false-negative rate (FNR), defined as the ratio of false negatives to the sum of false-negative and true positives.

In our modified HA routine, for each spectrum in the dataset a local search for nearby the assigned reference peaks is computed. If the distance of the peaks in the tested spectrum to the peaks in the reference is more than 0 and below a defined threshold (0.4 ppm for the F1 and 0.04 ppm for F2), then the peak is considered as a “moved” and its shift is added to the total sum of differences reported to the user for each spectrum. If the shift is above this threshold the peak is defined as “missing” and the total number of “missing” peaks is also presented to the user, thus indicating some interaction with the protein or an acquisition problem; such a spectrum is thus considered as an active upon user review. If there are no peaks moving, then the spectrum is marked as inactive.

In Table 1 is reported a side-by-side comparison of the performances between the analytical approach based on the customized Hungarian method and our ML-based discriminator.

**Table 1:** Comparison of HA and ML discriminators versus the user selection for each protein tested. FP and FN refer to false positives and false negatives respectively; TA and TI refer to predicted true actives and predicted true inactives. CP is the total number of correct predictions (TA + TI); accuracy is the correct prediction rate and is defined as CP over the number of the total experiments. FNR refers to the false-negative rate and is defined as the ratio between the number of FN to the sum of the TA and FN; FPR is the false-positive rate, defined as the ratio between the number of FP and the sum of the TI and FP. Sensitivity is calculated as 1 – FNR; specificity is calculated as TI over the sum of TI and FP. MCC is the Matthews correlation coefficient [31].

	Protein 1		Protein 2		Protein 3		Protein 4		Total	
Actives_Manual_	13		67		45		51		176	
Inactives_Manual_	321		212		519		383		1435	
Total	334		279		564		434		1611	

	HA	ML	HA	ML	HA	ML	HA	ML	HA	ML

Actives	1	28	53	73	6	63	97	128	157	292
Inactives	333	306	226	206	558	501	337	306	1454	1319
FP	1	20	15	11	6	40	74	95	96	166
FN	13	5	29	5	45	22	28	18	115	50
TA	0	8	38	62	0	23	23	33	61	126
TI	320	301	197	201	513	479	309	288	1339	1269
CP	320	309	235	263	513	502	332	321	1400	1395
Accuracy	0.96	0.93	0.84	0.94	0.91	0.89	0.76	0.74	0.87	0.87
FP (%)	0.30	5.99	5.38	3.94	1.06	7.09	17.05	21.89	5.96	10.30
FN (%)	3.89	1.50	10.39	1.79	7.98	3.90	6.45	4.15	7.14	3.10
Sensitivity	0.00	0.62	0.57	0.93	0.00	0.51	0.45	0.65	0.35	0.72
Specificity	1.00	0.94	0.93	0.95	0.99	0.92	0.81	0.75	0.93	0.88
FNR	1.00	0.38	0.43	0.07	1.00	0.49	0.55	0.35	0.65	0.28
FPR	0.00	0.06	0.07	0.05	0.01	0.08	0.19	0.25	0.07	0.12
MCC	−0.01	0.39	0.54	0.85	−0.03	0.37	0.20	0.28	0.29	0.49

While the overall accuracy of both HA and ML discriminators are comparable, the FNR is considerably lower for the ML-based analysis. This is a highly desired feature for an automatic discriminator because a high number of false negatives means that some of the active fragments may not be considered during the processing of the FBS data.

Our model had to cope with three major issues to make successful predictions; first, that protein spectra vary substantially from protein to protein, second, that the binding of a ligand results in different peak behavior depending on which specific residues it interacts with, and thirdly, that we have very imbalanced outcomes, with, in general, many more inactives than actives, but with the minority class the more interesting of the two for end-users.

From the user perspective, we aimed to create intuitive, reliable software to facilitate the rapid analysis of many spectra, and rigorously distinguish the actives from the inactives, to help the user optimizing their workflow and standardizing results.

### 4.2 Spectrum processing

To minimize the difficulty of resolving the first and second issues, we optimized the input representation passed to our machine-learning model by adopting a range of approaches from the field of computer vision and image analysis. This results in a fifteen element vector for each spectrum, representing the structural transformation between it and the spectrum provided as a reference.

Broadly, these can be divided into two approaches, descriptor-based and statistical. Beginning with the descriptor approaches, the histograms of oriented gradients (HOG) approach [32] describes the distribution of ‘edges’ (regions of high peak concentration) in an image, capturing information on local contours, silhouette, and some associated textural information. The local peak contour map is then binned, and compared to a similarly-prepared reference representation.

To account for the appearance and disappearance of peaks, we also register the translation of our query on the reference [33]. This approach determines the shifts that would be required to map one onto the other by cross-correlation, with a zero-error, zero-phase result indicating that they are identical. Rather than using the shifts themselves, we consider the error term which incorporates them, and the sum of the phase-difference. The phase-difference term takes the sum of the absolute position-wise differences between spectra.

The last of these is the oriented FAST and rotated BRIEF (ORB) [34] method for point-matching. It differs from the other methods discussed in that it attempts an explicit mapping between points in the reference and the query spectra, and can cope with an affine transformation from one to the other. We then calculate pairwise distances between the observed features and retain the largest ten of these to capture those points with most movement, which are likely those corresponding to the on-target binding, if present. In addition, we take the median of all ORB distance values to give a more general measure of the degree of peak-shifting between spectra.

**Fig. 6:**
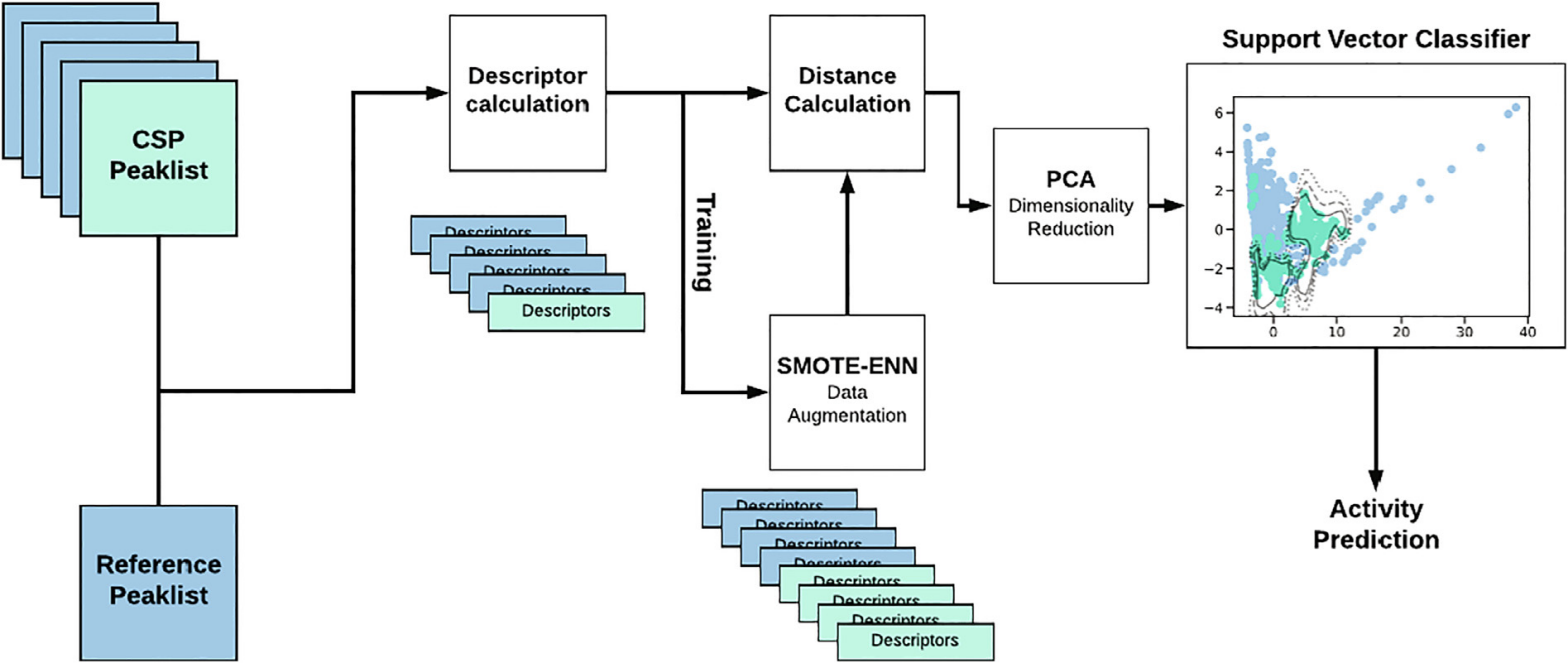
Overview of the ML discriminator approach. Given a set of CSP peak lists (prepared as discussed above), along with a chosen reference peak list, the program calculates a set of computer vision-based descriptors for each. During training, the provided peak lists have an associated label, indicating whether or not they are associated with a binding event. These labels are then used, in the first instance, to facilitate synthetic enhancement through the SMOTE-ENN data synthesis approach, essentially generating ‘fake’ examples of what a binding-associated peak list looks like. In both training and prediction modes, each peak list is then compared to the annotated reference, and the set of per-element distances used passed to a dimensionality-reduction approach (PCA). Finally, these reduced, comparative distance vectors are passed to a support-vector classifier (SVC). In the case of training, the associated labels are also provided to this algorithm, in which case it is directed to learn a maximally-separating hyperplane between the set of peak lists considered active, and those considered inactive. In addition, the kernel is directed to learn a calibrated probability, i.e. to produce probabilistic assessments of a given novel peak list indicating binding, given what it has seen before. In the prediction case, this ‘pseudo-probability’ is then returned to the end-user.

Coming from another perspective, we begin with the structural similarity index [35]. This is a highly noise-sensitive measure of the structural ‘integrity’ of our query spectrum compared to the reference. Its inclusion allows for the incorporation of information about the relative noisiness of our spectrum, and also a bulk-similarity measure. When compared to mean square error (MSE), a simple measure of the squared intensity difference, pixel-to-pixel, of two images, the structural similarity index produces a more human-consistent value, punishing images with a lot of Gaussian noise [36]. Similarly, we employ the well-established Hu moment estimation [37] to calculate the invariant properties of the reference and query spectra. These allow us to capture information about the localized dispersion of peaks in our frame of reference. We then take a simple paired-distance approach to compare the similarity of the two spectra. The final elements of this statistical approach that are included are the traditional normalized MSE and peak signal-to-noise ratio as absolute comparators, although their performance is somewhat lower than the aforementioned methods on most image-analysis tasks. As an additional simple metric, we include the difference in Jensen-Shannon entropy between the spectra (Fig. 6).

### 4.3 Machine-learning discriminator

From the machine-learning perspective, the imbalance in our data classes represents a significant challenge, with the regions of our descriptor space containing inactives considerably more densely populated than the rest. This also means that any machine-learning algorithm can perform relatively well by always predicting a spectrum as inactive, given their relative abundance. Two solutions to this problem are to weight our predictions, such that the model places equal importance on the relatively few actives as the many inactives, or data synthesis (also known as oversampling), where we create pseudo-actives, based on similarity in their descriptor representation. For training our model, we trialed both of these methods, ultimately deciding on the latter on the basis of performance.

The synthetic active spectra were generated combining synthetic minority over-sampling technique (SMOTE) [38] with edited nearest-neighbor under-sampling (ENN) [39] (SMOTE-ENN). SMOTE generates new data examples by considering pairs of active spectra, and generating synthetic data with descriptor vectors intermediate between the two samples. ENN cleans up the resulting data representation by removing points whose nearest neighbors have mixed classes. We used this to balance the number of experimental inactives and mixed experimental-synthetic active spectra.

To reduce the impact of highly-correlated data in our input representation, we undertook dimensionality reduction of the input by means of principal components analysis (PCA), projecting into a lower-dimensional space and identifying combinations of input features which capture most of the variance in the data. Given the impact of widely-varying units in estimating the contribution of each component, it was necessary to first normalize each element of the vectors by use of a robust scaler which scales according to the interquartile range. This method is somewhat more resilient in the presence of outliers than mean scaling.

As we wished to delineate a region of this space that captures inactive spectra, support-vector classification (SVC) was a natural choice. Briefly, this approach transforms a set of data that cannot be easily separated in the input space into a higher-dimensional space where it is possible, using a kernel approach. We utilized a radial basis function kernel, with heavy error penalization (C = 3), and enable classification probability estimation. This latter component is accomplished by Platt scaling, which fits a logistic regression model to the classification scores produced by the model. This allows us to return some measure of the model’s confidence about any given spectrum to the end-user, and therefore to rank them in a natural way. Our parameters were optimized so as to minimize the false-negative rate.

## 5 Conclusions and future development

Our method is capable of discriminating and recalling with good reliability the spectra which show sufficient change, promoting greater efficiency in screening analysis, and reduction of human bias induced by the repetitive nature of the task. While the program currently relies on third-party software for peak picking, future versions might directly utilize raw NMR time-domain data in order to create a comprehensive NMR analysis tool for FBDD. Allowing the user to adjust the intensity levels to their needs and set the thresholds for peak picking, would be a logical extension of the GUI. We also considered implementing functionalities to bridge the visualization module with available NMR data processing and visualization tools currently available, such as NMRPipe [40], PINT [41], NMRView [42], Sparky [43], and CCPNMR [27]. While the CSP Analyzer was designed to screen HSQC ^1^H-^15^N spectra, it could also be used for ^1^H–^13^C or any other bidimensional experiment. To facilitate this, a module for automatic recognition of the F1 and F2 dimensions could be added. We also considered the development of a KNIME [44] node for the CSP Analyzer which could then be interfaced with a general workflow for batch NMR data processing.

The backend utilizes several established computer vision approaches, coupled with Machine Learning, to achieve solid results in the recall of active-labeled spectra. Future work might replace this image-based approach with a model that can directly analyze waveform data [45], perhaps combined with some more direct means of determining which noise level can be safely ignored [46].

Overall, however, the relatively simple framework set out here sufficed to achieve good recall on a laborious task. It is uncertain how much of the gap from perfect recall to that achieved is owing to ambiguity and variation between experts in analysis of these spectra, but, in general, we could demonstrate that the speed of NMR FBDD data analysis can be greatly improved using our implementation of Machine Learning methods without a substantial decline in accuracy.

## 6 Materials and methods

### 6.1 NMR fragment-based screening

The in-house libraries of fragments were purchased from Thermo. Aqueous protein solutions of approximately 100 μM concentration with 10% D_2_O were added to 1.8 µL of fragment cocktails dissolved in deuterated DMSO in a 96-well plate with a total volume of 180 µL. Samples of 160 µL were then transferred to 3 mm NMR tubes using a Gilson liquid handling robot and So-fast HMQC or HSQC spectra were recorded at 298 K using Bruker Avance 600 MHz spectrometer equipped with a 5 mm QCI cryo-cooled probe head. A reference sample was made with d6-DMSO instead of fragment solution.

## Fundings

This work was supported by the European Union’s Framework Programme for Research and Innovation Horizon 2020 (2014–2020) under the Marie Skłodowska-Curie Grant Agreement No. 675555, Accelerated Early staGe drug dIScovery (AEGIS) for the economic support. This work is also supported by the Helmholtz Association Initiative and Networking Funds under project number ZT-I-0003.

## Author contributions

R.F. designed and wrote the code for the GUI and all the C# modules in the frontend application framework, R.B. wrote and validated the ML algorithm and built the Miniconda environment for the backend processing; C.A.S. expressed some of the proteins used in the NMR screening; R.F., C.A.S., and G.M.P. recorded and processed NMR spectra. M.S., G.S., and G.M.P. provided assistance and technical supervision. R.F., R.B., and C.A.S. wrote the manuscript and M.S., G.S., and G.M.P. helped with corrections and scientific advice.

## Declaration of competing interest

The authors declare no conflict of interest.

## CRediT authorship contribution statement

**R. Fino:** Conceptualization, Methodology, Software, Validation, Formal analysis, Investigation, Data curation, Writing - original draft, Writing - review & editing, Visualization. **R. Byrne:** Conceptualization, Methodology, Software, Validation, Formal analysis, Data curation, Writing - original draft, Writing - review & editing, Visualization. **C.A. Softley:** Resources, Investigation, Data curation, Writing - original draft, Writing - review & editing. **M. Sattler:** Supervision, Project administration, Writing - review & editing, Funding acquisition. **G. Schneider:** Supervision, Project administration, Writing - review & editing, Funding acquisition. **G.M. Popowicz:** Conceptualization, Methodology, Software, Writing - review & editing, Supervision, Project administration, Funding acquisition.
